# Circulating biomarkers during progression to type 1 diabetes: A systematic review

**DOI:** 10.3389/fendo.2023.1117076

**Published:** 2023-02-03

**Authors:** Ekua W. Brenu, Mark Harris, Emma E. Hamilton-Williams

**Affiliations:** ^1^ School of Medicine, University of Notre Dame, Sydney, NSW, Australia; ^2^ Endocrinology Department, Queensland Children’s Hospital, South Brisbane, QLD, Australia; ^3^ Frazer Institute, The University of Queensland, Woolloongabba, QLD, Australia

**Keywords:** biomarkers, islet autoimmunity, type 1 diabetes, disease progression, miRNA, systematic review, immune markers

## Abstract

**Aim:**

Progression to type 1 diabetes (T1D) is defined in stages and clinical disease is preceded by a period of silent autoimmunity. Improved prediction of the risk and rate of progression to T1D is needed to reduce the prevalence of diabetic ketoacidosis at presentation as well as for staging participants for clinical trials. This systematic review evaluates novel circulating biomarkers associated with future progression to T1D.

**Methods:**

PubMed, Ovid, and EBSCO databases were used to identify a comprehensive list of articles. The eligibility criteria included observational studies that evaluated the usefulness of circulating markers in predicting T1D progression in at-risk subjects <20 years old.

**Results:**

Twenty-six studies were identified, seventeen were cohort studies and ten were case control studies. From the 26 studies, 5 found evidence for protein and lipid dysregulation, 11 identified molecular markers while 12 reported on changes in immune parameters during progression to T1D. An increased risk of T1D progression was associated with the presence of altered gene expression, immune markers including regulatory T cell dysfunction and higher short-lived effector CD8^+^ T cells in progressors.

**Discussion:**

Several circulating biomarkers are dysregulated before T1D diagnosis and may be useful in predicting either the risk or rate of progression to T1D. Further studies are required to validate these biomarkers and assess their predictive accuracy before translation into broader use.

**Systematic review registration:**

https://www.crd.york.ac.uk/prospero, identifier (CRD42020166830).

## 1 Introduction

Type 1 diabetes (T1D) is a multifactorial, heterogeneous, and polygenic autoimmune disorder characterised by the destruction of pancreatic beta cells resulting in loss of insulin secretion and consequent hyperglycaemia. Presently, the global incidence and prevalence of T1D are increasing with an estimated incidence rate of 15 per 100,000 people and prevalence of 9.5 per 10,000 (1). T1D is diagnosed predominantly in children younger than 15 years (2), however, it may also occur in adulthood (3). TID is a clinical diagnosis based on blood glucose concentration, HbA1c, C-peptide concentrations and islet-specific autoantibodies in combination with typical symptoms (4). However, these symptoms are preceded by a period of ongoing autoimmunity involving immune activation and destruction of insulin secreting beta cells. While this phase of autoimmunity is clinically silent, it represents an opportunity to identify individuals at the greatest risk of developing T1D (5). Identification of these individuals would have multiple benefits including reducing the risk of diabetic ketoacidosis at diagnosis and allowing identification of subjects for new preventative interventions.

The risk factors for T1D are both genetic and environmental. Specific human leukocyte antigen (HLA) class II alleles are associated with increased risk, for example about 30-50% of children with T1D have the combined DR3-DQ2 and DR4-DQ8 haplotype (6). Environmental risk factors for T1D have not been clearly defined but may include nutrition, gut microbiota, antibiotic use, viral infections, route of birth, and the intrauterine and maternal environment (7). These genetic and environmental risk factors initiate a sequence of events that activate autoreactive T cells and subsequent destruction of beta cells, with eventual loss of insulin secretion (8). Prior to diagnosis, autoimmune reactivity is currently identified by the emergence of autoantibodies against proteins linked to the beta cell secretory pathway (9). The presence of islet autoantibodies and high-risk HLA genotypes are well described risk factors for T1D (10) and individuals possessing high-risk HLA genotypes advance to autoantibodies more often compared with individuals with moderate-risk genotypes (11).

Recently, the progression to a clinical diagnosis of T1D has been classified into three stages: Stage 1- a presymptomatic phase characterised by seroconversion (≥ 2 islet autoantibodies), and normoglycaemia; Stage 2- presymptomatic phase characterised by seroconversion and dysglycaemia; and Stage 3 - symptomatic stage characterised by islet autoimmunity and hyperglycaemia (12). The time spent in the presymptomatic stages of T1D varies extensively. Although autoantibodies have proven reliable predictors of T1D, additional biomarkers linked to the rate of progression through these presymptomatic stages of T1D would help identify underlying mechanistic pathways. Accurate circulating biomarkers are highly useful for disease prediction, being relatively easy to access (13). The identification of predictors of clinical T1D may help to elucidate disease pathogenesis and inform the use of preventative therapy. This systematic review evaluates identified circulating predictors of progression from presymptomatic stages to the clinical diagnosis of T1D.

## 2 Methods

This systematic review was registered on PROSPERO (CRD42020166830) and reported in accordance with the Preferred Reporting Items for Systematic Reviews and Meta-Analyses (PRISMA). The study inclusion dates were amended to 1 January 2010 – 31 August 2022.

### 2.1 Literature search

Original research articles selected from PubMed, Ovid and EBSCO and published between 2010 and 2022 have been included in this study. Articles were selected using the following search terms: (type 1 diabetes) AND (prediction/predicting/predict/predictor/progression/biomarker/islet autoimmunity). The term ‘Type 1 diabetes’ was searched in the title only. Other search terms including prediction, predicting, predict, predictor, progression, progressor(s), biomarker(s), disease risk, indicator(s), susceptibility, and prognosis were searched individually in combination with Type 1 diabetes in the search field tags title and text word (**Supplementary methods**).

### 2.2 Selection criteria

The full text of titles and abstracts were reviewed to determine eligibility. Studies were deemed eligible using the following inclusion criteria: (1) observational studies (2) analysis of circulating markers predicting onset or progression to T1D or islet autoimmunity; (3) population size of at least 20 participants; (4) participants younger than 20 years old; and (5) participants with HLA DR or/and DQ haplotypes associated with T1D and one or more of the following; a family history of T1D, confirmed islet autoantibodies, abnormalities in glucose metabolism, deficient beta cell function, and T1D. The following studies were excluded (1) reviews, systematic reviews, meta-analyses, studies primarily reporting outcomes from randomized controlled clinical trials, case reports, case series, conference papers or editorials; (2) abstracts where full text components were not available; (3) animal models of T1D; (4) participants older than 20 years; (5) participants with type 2 diabetes; (6) pregnant participants; (7) studies focussed solely on autoantibodies and (8) non-English articles. The search and screening process was objectively performed by one reviewer (EWB) and reviewed by another (EHW). Randomised controlled trials were excluded from this current study as this study focuses on the natural progression of disease and not the response to interventions. Studies with more than 20 participants were chosen as they had more statistical power. Given that T1D is a heterogeneous disease, we chose to focus on participants aged <20 years to minimise the effects of other comorbidities and to allow for identification of markers that may be present early in the disease process prior to adulthood.

### 2.3 Quality assessment of studies

All selected articles were assessed for quality of research using the Critical Appraisal Skills Programme (CASP) for cohort studies and case control studies (CASP, 2018). Markers of quality included study design, methods, and duration of study (**Supplemental Tables S1, S2**).

### 2.4 Data extraction

For all studies meeting the inclusion criteria the data extracted was transferred into an excel spreadsheet. The following information was retrieved from each study: first author, date of publication (year), country or region, diagnostic criteria for T1D, number of cases and controls, type of cases, age at diagnosis or age at sample collection, presence or absence of autoantibodies, male to female ratio and results. The primary outcome was predictors of progression to T1D, where measures of prediction were defined as: (1) gene expression; (2) proteins and lipids; and (3) and immune markers (cells and cytokines). Data extraction from eligible full text articles was performed by one reviewer and reviewed by two reviewers (EHW and MH). A meta-analysis was not possible due to the high degree of heterogeneity between the studies.

## 3 Results

### 3.1 Characteristics of the studies

**Figure 1:**
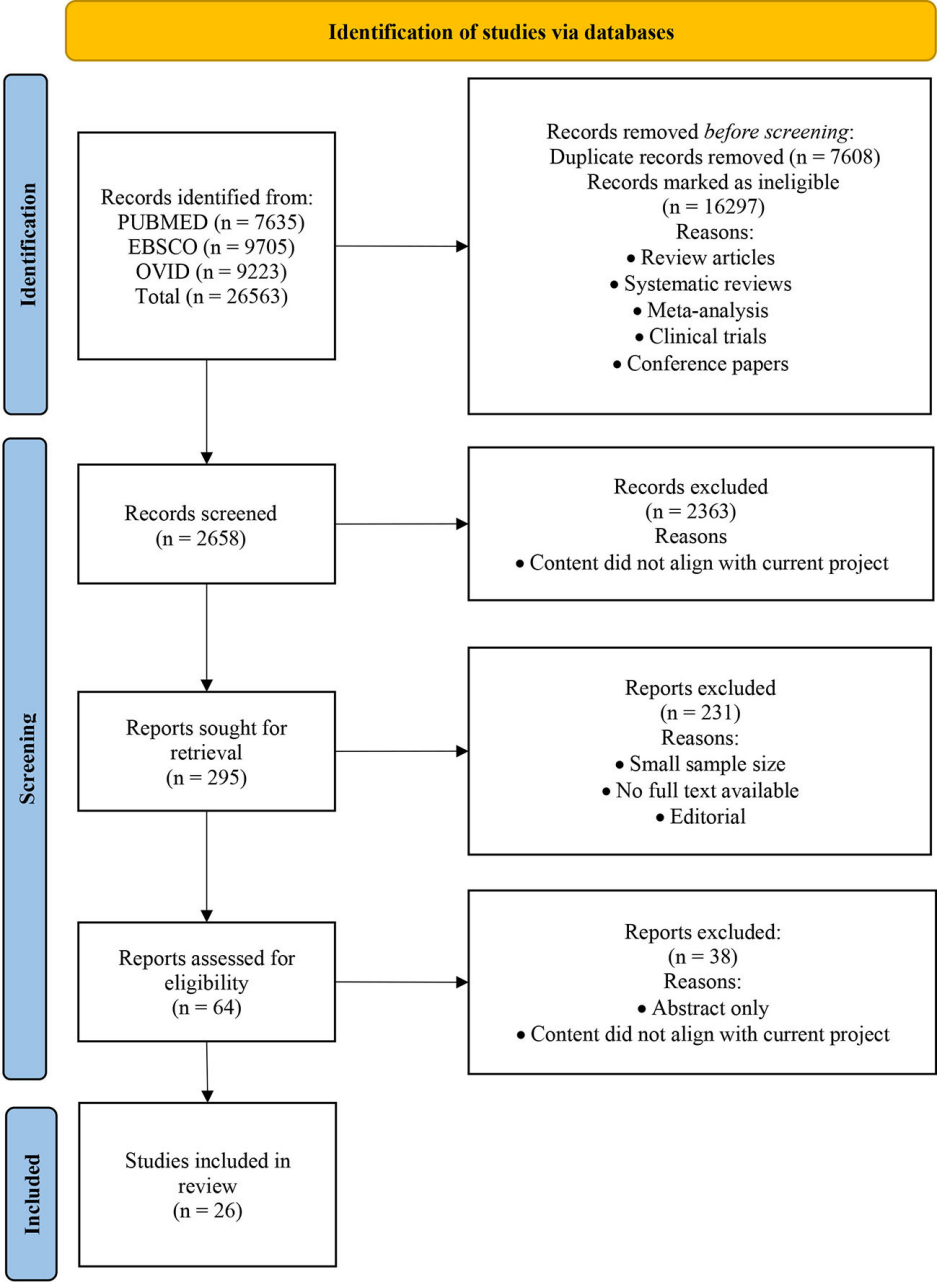
PRISMA Flow chart of selection of studies for the systematic review.

Initially, 26546 articles were identified following evaluation of titles and text words (**Figure 1**). Following the inspection process outlined in **Figure 1**, 26 articles were chosen for the systematic review. These articles comprised 17 cohort and 9 case control studies (10, 14–36). The characteristics of the selected studies and study participants are presented in **Table 1**. Among the 26 studies, four did not report on the age at seroconversion or age at the time of sample collection. The 17 cohort studies, included the TrialNet pathway to prevention study (TrialNet), Diabetes prediction and prevention study (DIPP), Diabetes Autoimmunity study in the young (DAISY), Primary Prevention of Type 1 Diabetes in Relatives at Increased Genetic Risk (BABYDIET), German BabyDiab study (BABYDIAB), Pathogenesis of type 1 diabetes-testing the hygiene hypothesis (DIABIMMUNE) and the environmental determinants of diabetes in the Young (TEDDY) cohort studies (10, 20–23, 25–29, 31, 32, 34–36). The 26 selected studies used in the current systematic review were categorised according to marker type with 5 focused on protein and lipid markers, 12 on immunological markers and 11 on molecular markers.

**Table 1:** Characteristics of studies included in the systematic review.

Authors	Year	Study method	Country	Diagnostic criteria	Cases (n)	Type of participants	Age (median/ mean)	Healthy Control (n)	Age (median/mean)
Arif et al. (14)	2014	Case control study	UK	American diabetes association	33	ROT1D	11 (5–16)	72	13 (6–16)
Ferreira et al. (15)	2014	Case control study	UK	American diabetes association	178	ROT1D=49 Ab+=109	12 (6–34) 1.5 (0.2-9.1)	118	43 (29–61)
Garavelli et al. (16)	2020	Case control study	Italy	WHO criteria	150	ROT1D=88 ROT1D (12 months) =32 ROT1D (24 months) =30	9 (4) 12 (4) 12 (4)	47	
Glisic et al. (17)	2012	Case control study	USA	WHO criteria	52	ROT1D=29 IAb+=23	12.05 (0.91) 27.58 (3.16)	35	30.2 (2.75)
Hamari et al. (18)	2016	Case control study	Finland	WHO criteria	72	Single IAb+=20 Multiple IAb+ =22 ROT1D=30	7.4 (0.6-15.4) 9.7 (2.4-14.7) 7.6 (0.8-15.7)	72	7.7 (1.5-15.4) 9.7 (2.8-15.4) 7.8 (1.5-15.0)
Han et al. (19)	2011	Case control study	USA	American diabetes association criteria	47	ROT1D n=29 at risk n=18	15.3 (8.2) 14.8 (12.4) 39.3 (13.8)	80	90.0 (10.2)
Harms et al. (20)	2018	Cohort – TrialNet	USA	American diabetes association criteria	133	IAb- =25 IAb+ =54 Ab+ NP =26 Ab+ P = 28	12.2 (2.7) 10.9 (4.6) 10.6 (4.0) 11.3 (5.1)		
Ihantola et al. (21)	2018	Cohort – DIPP	Finland Sweden Denmark	WHO criteria	168	ROT1D n= 40 IAb+ n=39 IAb- n=89	8.6 (3.9) 8.7 (4.6) 9.2 (3.9)		
Jin et al. (22)	2014	Cohort – DAISY	USA	American diabetes association	104	IAb+ P =39 IAb+ NP =65	7.4 (1.74-15.02) 9.05 (1.55-45.08)		
Lamichhane et al. (23)	2018	Cohort study DIPP	Finland Sweden Denmark	WHO criteria	80	T1D n=40 IAb+ n=40 IAb- n=40	1.34 (0.58) 3.05 (2.50)		
Marchand et al. (24)	2016	Case control study	France	Not stated	22	ROT1D	9.81 (3.59)	10	9.9 (2.34)
Mehdi et al. (10)	2018	Cohort - BABYDIET & DIPP	Germany Finland	American diabetes association criteria	92	IAb+ =25 IAb- =67			
Nielsen et al. (25)	2012	Cohort study	Denmark	Not stated	404	ROT1D		151	6.7-13.7
Oling et al. (26)	2012	Cohort – DIPP	Finland	WHO criteria	96	T1D =31 Ab+ = 65		93	
Pflueger et al. (27)	2011	Cohort – BABYDIAB	Germany	American diabetes association criteria	70	IAb+=35 IAb-=35	9.1 (8.2-13.3) 9.7 (8.1-11.2)		
Reinert-Hartwall et al. (28)	2015	Cohort – DIABIMMUNE	Finnish Estonian	American diabetes association	159	IAb+ = 38 IAb- =80 T1D = 15	6.7 (3.7) 4.8 (2.3) 8.3 (5.5)		
Santos et al. (37)	2022	Case control study	Brazil	American diabetes association criteria	110	IAb+ = 25 ROT1D =30	11.79 (9–19) 13 (9.9-18.6)	29	14.5 (10-18.9)
Salami et al. (29)	2018	Cohort – TEDDY	Sweden	American diabetes association criteria	448	IAb+ =72 IAb- = 376	8.8 (4.9-11.5) 7.5 (4.3-11.5)		
Sen et al. (30)	2020	Cohort – DIPP	Finland	WHO criteria	61	IAb+ P = 34 IAb+ NP=27	4.42 (2.54)	10	
Simmons et al. (31)	2019	Cohort – TrialNet	USA	American diabetes association criteria	57	T1D	7-17		
Snowhite et al. (32)	2017	Cohort – TrialNet	USA	American diabetes association criteria	450	IAb-=150 IAb+ =150 IAb+ NP =111 IAb+ P =39	11.1 (3.7) 10.9 (3.7) 10.8 (3.7) 11.2 (3.5)	50	24.9 (4.7)
Starosz et al. (38)	2022	Case control study	Poland	Not stated	60	ROT1D =60	11 (9.0-13.5)	31	13.5 (9.5-14.5)
Vecchione et al. (33)	2020	Cohort – TrialNet	Italy	American diabetes association criteria	82	IAb-=24 IAb+=10 Stage 1 = 9 Stage2 = 10 T1D=29	11 (5–17) 11 (5–17) 10 (7–13) 13 (5–17) 12 (4–17)		
Viisanen et al. (34)	2019	Cohort –DIPP	Finland	WHO criteria	150	T1D =74 IAb+ =76 IAb- =180	7.8 (4.1) 9.4 (4.7) 8.8 (4.0)		
von Toerne et al. (35)	2017	Cohort – BABYDIET & BABYDIAB	Germany	American Diabetes Association	2347	Multiple IAb+=124 IAb+ P=82			
Waugh et al. (36)	2017	Cohort – DAISY	USA	American diabetes association criteria	50	T1D = 25 IAb+ =25	10.5 12.6	25	10.9

Islet autoantibody (IAb), Progressors (P), Non-progressors (NP), Recent or newly diagnosed T1D (ROT1D), World Health Organisation (WHO).

### 3.2 Immune cell subsets and progression to T1D

**Figure 2:**
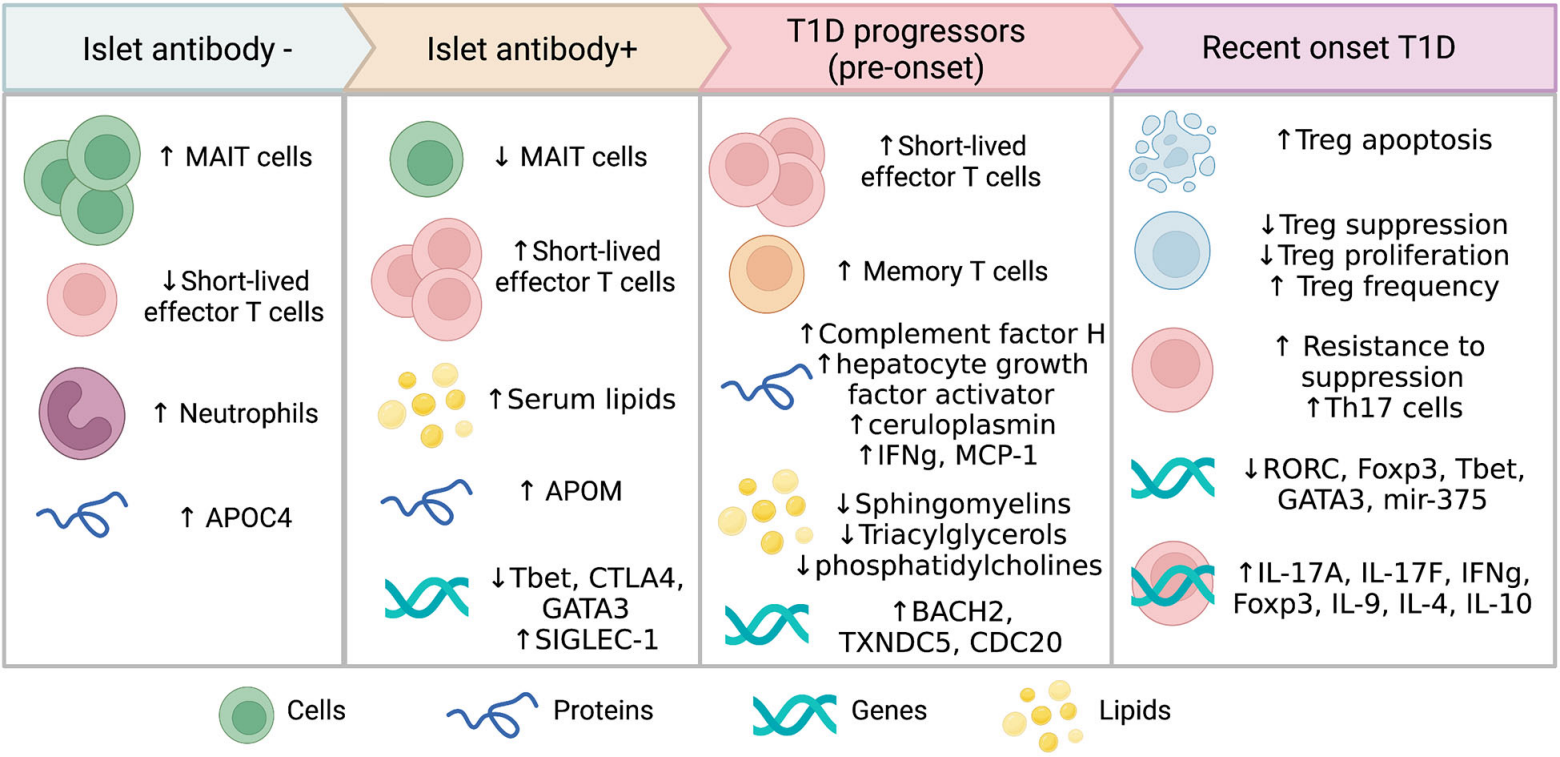
Circulating biomarkers associated with different stages of T1D progression.

Twelve studies identified alterations in the function and phenotype of different immune cell populations linked to progression to T1D or seroconversion (**Table 2**). Most of the immune studies focused on T cell subsets and their phenotype, with the changes observed varying depending on the disease stage (**Figure 2**).

**Table 2:** Immunological markers linked to progression to T1D or islet autoimmunity.

Authors	Year	Type of participants	Sample type	Immune markers
**Starosz et al., (** 38 **)**	2022	ROTID vs. controls	Peripheral blood	↑CD25^+^CD127^-^FOXP3^+^ Tregs ↑Tregs/Th17 ↑IL-10
**Vecchione et al., (** 33 **)**	2020	T1D vs. ROT1D	Peripheral blood	↑Follicular Tregs (CXCR5^+^FOXP3^+^) ↑Conventional Tregs (CXCR5^-^Foxp3+) ↑PD-1 on Tregs
**Viisanen et al., (** 34 **)**	2019	ROTID vs. controls	Peripheral blood	↑Total and naive Treg ↑CD25^+^FOXP3^low^ memory Tregs ↑CD25^low^CD127^low^FOXP3^+^ Tregs ↓Ki67+ Tregs ↓IFN-γ producing memory Tregs
**Ihantola et al., (** 21 **)**	2018	ROTID vs. IAb+	Peripheral blood Plasma	↑Teff resistance to Treg suppression Faster STAT3 activation on TCR stimulation
**Salami et al., (** 29 **)**	2018	IAb+ vs IAb-	Peripheral blood	↓White blood cells ↓Neutrophils ↓Haemoglobin ↓Haematocrit ↓ Red blood cells
**Harms et al., (** 20 **)**	2018	IAb+ vs IAb- IAb+ progressors to T1D vs non-progressors	Peripheral blood	↓MAIT cells (mucosal associated invariant T cells) ↓CD45RA^+^CCR7^dim^CD8^+^T cells ↑SLEC [short lived effector-like CD8^+^T cells (CD57^+^CD28^-^CD127^-^CD8^+^T cells)] ↓CD127^bright^ memory CD4^+^T cells ↓CD127^dim^CD4^+^T cells (Treg like cells)
**Waugh et al., (** 36 **)**	2017	IAb+ Progressors to T1D vs IAb+ non-progressors IAb+ vs. controls	Serum	↑CCL2 (MCP-1) and IFNγ in T1D progressors ↓CCL2 at seroconversion ↑CXCL10 (IP-10) in IAb+
**Reinert-Hartwall et al., (** 28 **)**	2015	T1D vs IAb+ IAb+ vs. IAb-	mRNA levels in antiCD3 and antiCD28 stimulated cells PBMCs or Activated Th17 cells	In antiCD3 and antiCD28 stimulated cells: ↑IL-17A ↑IL-17F ↑FOXP3 ↑IFNγ ↑IL-9 In Th17 cells: ↑IFNγ/IL-17
**Arif et al., (** 14 **)**	2014	ROTID	Peripheral blood	↑IL-10 in response to pro-insulin, insulin, GADA and IA-2
**Ferreira et al., (** 15 **)**	2014	IAb+ (before and after seroconversion) vs. IAb-	Peripheral blood monocytes	↑SIGLEC-1 (CD169)
**Glisic et al., (** 17 **)**	2012	ROT1D vs. HLA risk matched subjects	Peripheral blood	↑Treg apoptosis ↓Treg function
**Oling et al., (** 26 **)**	2012	T1D group vs. IAb+		↑Reactivity to GAD65_555-567_ by memory T cells

IAb, islet autoantibodies; LST1D, long-standing type 1 diabetes; ROT1D, Recent onset type 1 diabetes; GADA, Glutamate decarboxylase 65 autoantibodies; IA2A, insulinoma associated antigen 2 autoantibodies.

#### 3.2.1 T cell phenotype alterations prior to T1D onset

Seroconverted participants had decreased frequencies of mucosal associated invariant T cell (MAIT) and CD127^dim^CD4^+^ regulatory T cells (Tregs) compared with islet antibody negative (Ab-) participants. An increased ratio of short-lived effector CD8^+^ T cells (SLEC) T cells to MAIT cells as well as SLEC : CD127^dim^ cells was found in all seroconverted individuals (progressors and non-progressors) compared with Ab- participants (20). The increase in SLEC was highest amongst seroconverted individuals that later progressed to T1D (20). The SLEC (CD127-, CD27-, CD57+, CD28-) phenotype indicates short-lived, terminally differentiated cells undergoing chronic stimulation with cytotoxic potential. Non-progressors showed reduced CD45RA^+^CCR7^dim^ memory CD8^+^T cells and reduced CD127^bright^ memory CD4^+^T cells compared with Ab- participants (20).

#### 3.2.2 Impaired Treg regulatory function during progression to T1D

In recent-onset T1D (ROT1D), Glisic et al., reported no change in the number of Tregs but increased Treg apoptosis and reduced suppressive function compared with healthy controls, particularly in those with high-HLA risk (17). Similarly, during the first year after diagnosis Th17 and activated Tregs were elevated in some children compared with those with early beta cell autoimmunity (28, 38). Viisanen et al. found that ROT1D individuals had increased CD25^+^FOXP3^low^, CD25^low^CD127^low^FOXP3^+^ and total Tregs, due to an increase in naïve Tregs in comparison to healthy subjects (34). There was also a decrease in IFN-γ^+^ memory Tregs and Ki67^+^ proliferative Tregs at disease onset compared with controls (34). In another study, both follicular helper Tregs (CXCR5^+^FOXP3^+^) and conventional Tregs (CXCR5^-^FOXP3^+^) were high in ROT1D compared with Ab- controls, owing to high expression levels of FOXP3^+^ in CD4^+^ cells (33). However, in the same study, programmed cell death-1 (PD-1) expression, an immunoregulatory marker linked to T cell tolerance (39), was significantly diminished in Tregs from ROT1D and at-risk individuals compared with Ab- controls (33). Recently, Starosz et al. reported an increase in both Treg frequency and in the ratio of Treg to Th17 cells (38). While Tregs populations were either increased or unchanged in frequency in several studies (17, 33, 34, 38), effector T cells were found to be more resistant to Treg suppression in ROT1D when compared with multiple autoantibody positive subjects (21). Together, these data suggest an important role for a diminishment in Treg suppression of effector T cells at the time of T1D onset.

### 3.3 Protein and lipid markers predicting progression to T1D

Dysregulation in proteins and lipids were also observed in individuals progressing to T1D (**Table 3**). Islet autoantibody positive children had higher serum lipids compared to those children without islet autoantibodies (27). Three metabolic peptides were found to predict future progression to T1D including, complement factor H, hepatocyte growth factor activator and ceruloplasmin (35). Lipids such as sphingomyelins, triacylglycerols and phosphatidylcholines as well as methionine and hydroxyproline were reduced in peripheral blood mononuclear cells (PBMC) or plasma from individuals who progressed to T1D compared with controls (23, 30). These studies suggest that multiple factors may exert a differential effect on lipid levels during disease progression.

**Table 3:** Protein and lipid markers associated with progression to T1D.

Authors	Year	Type of participants	Protein and lipid markers
**Sen et al., (** 30 **)**	2020	IAb+ progressors to T1D vs. non-progressors	↓Lipid levels in PBMCs including phosphatidylcholines, sphingomyelins, ceramides, cholesterol esters, lysophosphatidylcholines, phosphatidylethanolamines and triacylglycerols
**Garavelli et al., (** 16 **)**	2020	ROT1D vs. healthy controls	↑soluble leptin receptor, ↑Osteoprotegerin ↓Myeloperoxidase
**Lamichhane et al., (** 23 **)**	2018	IAb+ progressors to T1D, IAb+ non-progressors and IAb-	↓Plasma lipid levels including sphingomyelins, triacylglycerols and phosphatidylcholines in progressors
**Von Toerne et al., (** 35 **)**	2017	IAb+ vs. IAb-	26 serum proteins differed with ↑APOM and ↓APOC4 best discriminating IAb+ Hepatocyte growth factor activator (↑, complement factor H (↑ and ceruloplasmin (↓predicted shorter time to T1D progression
**Pflueger et al., (** 27 **)**	2011	IAb+ vs. IAb-	↓Amino acid metabolites: Methionine and hydroxyproline ↑Lipid levels: polyunsaturated fatty acid-containing phosphatidylcholines, specific triacylglycerols

IAb, islet autoantibodies; ROT1D, recent-onset type 1 diabetes.

### 3.4 Messenger RNA gene expression before and after progression to T1D

Differential expression of genes in blood cell populations may provide insights into the function of immune cells and metabolic pathways as autoimmunity develops. Several genes were observed to be differentially expressed in ROT1D, in individuals progressing to T1D and even very early prior to seroconversion (**Table 4**; **Figure 2**) (10, 15, 18, 19, 22, 24, 25, 31, 32, 37). A signature of 56 genes inducible with type-1-interferon including *SIGLEC-1* was found to be upregulated prior to the development of autoantibodies in at-risk children compared with controls (15). As islet autoimmunity progressed, reduced expression of several transcription factors involved in T-cell differentiation were observed in whole blood RNA from individuals with multiple islet autoantibodies (*T-bet*, *CTLA-4* and *GATA3)* compared with Ab- control children (18). Higher expression of *BACH2*, *TXNDC5 and CDC20*, genes that regulate lymphocytes and cell cycle progression, were found in islet autoantibody+ children that later progressed to T1D compared with non-progressors (22). This suggests that altered gene expression profiles occur amongst blood immune cells prior to T1D onset and these may be useful for prediction of future diagnosis.

**Table 4:** Gene expression markers linked to T1D progression.

Authors	Year	Type of participants	Sample type	miRNA and mRNA genes
**Santos et al., (** 37 **)**	2022	IAb+ vs. ROT1D vs. health control	Serum	17 miRNAs were deregulated in IAb+ and 5 in ROT1D compared to healthy controls
**Garavelli et al., (** 16 **)**	2020	ROT1D vs. healthy controls	Plasma	27 of 60 miRNAs were modulated including 18 of 27 from the miR-23~27~24 clusters
**Simmons et al., (** 31 **)**	2019	ROT1D vs. IAb+	Serum DNA	↑unmethylated:methylated *INS* ratio
**Mehdi et al., (** 10 **)**	2018	Progressors to IAb+ vs. IAb-	Whole blood (DIPP), PBMC (BABYDIET)	Predictors of seroconversion *ADCY9, PTCH1, MEX3B, ZNF714, TENM1, PLEKHA5* with HLA risk score
**Snowhite et al., (** 32 **)**	2017	IAb+ Progressors to T1D vs. non-progressors	Serum	↑*miR-21-3p*, ↑*miR-29a-3p*, ↑*miR-424-5p*
**Hamari et al., (** 18 **)**	2016	IAb+ vs. ROT1D	Whole blood	↓*T-bet*, ↓*GATA*3, ↓ *RORC*, ↓*FOXP3*
**Marchand et al., (** 24 **)**	2016	ROT1D vs IAb-	Serum	↓*miR-375*
**Jin et al., (** 22 **)**	2014	IAb+ Progressors to T1D vs. non-progressors	Whole blood	↑*BACH2*, ↑*IGLL3*, ↑ *EIF3A*, ↑*CDC20*, ↑ *TXNDC5*
**Ferreira et al., (** 15 **)**	2014	IAb+ (before and after seroconversion) vs. IAb-	PBMC	↑type-1 IFN gene signature (includes 56 IFN-inducible genes)
**Nielsen et al., (** 25 **)**	2012	ROT1D vs. IAb+	Serum	↑*miR-181a*, ↑*miR-24*, ↑*miR-25*, ↑*miR-210*, ↑*miR-26a*, ↑*miR-152*, ↑*miR-30a-5p*, ↑*miR-148a*, ↑*miR-200a*, ↑*miR-29a*, ↑*miR-27b*, ↑*miR-27a*
**Han et al., (** 19 **)**	2011	ROT1D and IAb+ vs. healthy controls	Whole blood	↑*IFN-γ*, ↑*IL-4*, ↑*IL-10* in ROT1D vs IAb+ ↓g*ranzyme B*, ↓*TNFα*, ↓*IFN-γ* in IAb+ vs healthy controls

ADCY9, Adenylate cyclase type 9; GATA3, GATA binding protein 3; IAb, islet autoantibody; MEX38, Mex-3 RNA Binding Family Member B; miRNA, MicroRNA; PLEKHA5, Pleckstrin Homology Domain Containing A5; PTCH1, Protein patched homolog 1; ROT1D, recent-onset type 1 diabetes; TENM1, Teneurin Transmembrane Protein 1; TENM1, Teneurin Transmembrane Protein 1; ZNF714, Zinc finger 714.

In ROT1D, the expression of T cell specific transcription factors including *RORC*, *FOXP3*, *T-bet* and *GATA3* were reduced compared with Ab- control children (18). However, mRNA levels of several genes (*IL-17A*, *IL-17F*, *IFN-γ*, *IL-9* and *Foxp3*) linked to T cell effector function or regulation were increased in PBMC stimulated with anti-CD3 and anti-CD28 from individuals with ROT1D compared with Ab- controls (28). Cytokine genes were also upregulated in non-activated PBMCs from ROT1D compared to islet autoantibody+ individuals (*IFN-γ, IL-4* and *IL-10*) (19). Of interest, higher unmethylated INS DNA, a marker of beta cell death, was observed in serum from ROT1D participants compared with islet autoantibody+ participants (31). The above biomarkers suggest that multiple physiological processes are disrupted during progression to T1D.

### 3.5 MicroRNA expression and progression to T1D

Differential expression of microRNAs (miRNA) with varying roles in metabolic and immune function were observed in several studies. Importantly, 11 miRNAs were upregulated and 6 were down regulated in islet autoantibody+ participants compared with healthy controls (37). In contrast, only 4 miRNA were upregulated and one downregulated in participants with ROT1D. Increased expression of *miR-23a-3p*, *miR-23b-3p*, *miR-24-3p*, *miR-27a-3p*, *miR-27b-3p*, *miR-21-3p*, *miR-29a-3p* and *miR-424-5p* were observed in islet autoantibody+ participants who progressed to T1D compared with healthy controls (16, 32). A downregulation of *miR-375* was observed in individuals with ROT1D compared with healthy controls (24), while *miR-152*, *miR-30a-5p*, *miR-181a*, *miR-24*, *miR-148*, *miR-200*, *miR-210a*, *miR-27a*, *miR-29a*, *miR-27b*, *miR-26a* and *miR-25* were upregulated in ROTID participants compared with healthy controls (25). Of interest, *mir-200a-3p* was consistently upregulated in both islet autoantibody+ and ROT1D and *miR-16* was reduced in both groups (37). Several of these miRNAs have been linked to beta cell apoptosis and damage (40, 41), suggesting an important role of miRNAs in the pathogenesis of T1D.

## 4 Discussion

Biomarkers predicting progression to T1D are urgently needed and represent an emerging area of research interest. This systematic review of case control and cohort studies summarises important circulating biomarkers in individuals progressing to clinical T1D (**Figure 2**). These circulating biomarkers comprise immunological, metabolic, and molecular factors. Biomarkers with concordant evidence from multiple studies included increased Treg numbers but decreased functionality, while other markers such as cytokine expression patterns and lipid levels were discordant. Collectively, these biomarkers suggest that prior to diagnosis immune dysfunction accelerates with increased effector cell function and lack of immune suppression, coinciding with beta cell functional decline. However, there are still unknown mechanistic pathways involved in this critical period.

Several markers of beta cell destruction were identified in the current study. Beta cells are the main producers of unmethylated *INS* and a higher unmethylated: methylated *INS* is observed during progression to T1D (31). The presence of circulating unmethylated *INS* DNA is a marker for beta cell death and reduction in insulin which accelerates at the symptomatic stage of the disease (42). *MiR-375* is highly expressed in islet cells and regulates development of islet cell mass (alpha and beta) (43). In animal models, deficiency in *miR-375* islet cells results in persistent hyperglycaemia due to alteration of pancreatic α-cell function, including elevated α cell mass (24) and a concomitant decrease in beta cell mass due to upregulation of destructive growth factor genes including apoptosis-inducing factor, mitochondrion associated 1 (*Aifm1*), caveolin1 (*Cav1*), complement component 1 q subcomponent binding protein (*C1qbp*), cell adhesion molecule 1 (*Cadm1*), eukaryotic elongation factor 1 epsilon 1 (*Eef1e1*), HuD antigen (*HuD*), inhibitor of DNA binding 3 (*Id3*), Ras-dexamethasone-induced-1 (*Rasd1*), and regulator of G protein signalling 16 (*Rgs16*) (43, 44). Increased expression of *miR-29* and *miR-200a-3p* in beta cells promotes destruction of beta cells and progression to T1D (41, 45). Circulating *miR-25* increases residual beta cell function and glycaemic control (25) and more recently it has been shown to suppress translation of *INS* gene and decreased insulin production (46).

Importantly, Tregs are among the key cells implicated in the pathogenesis of T1D and polymorphisms in *IL2RA, CTLA4, PTPN2* and *PTPN22* have been shown to contribute to alterations in Treg function in T1D (8). In addition, CD8^+^T cells and other effector cells are involved in pancreatic insulitis prior to clinical diagnosis (47). The presence of these cells and SLEC cells suggest increased effector cell activity, and changes in effector cell to Treg ratios prior to disease onset. Differential expression of genes involved in the regulation of immune cell biology, are likely to explain the alterations in immune function in T1D (48). Notably, dysregulation in T cell transcription factors affects multiple T cell functional pathways. The increase in Treg numbers after T1D onset seems counter-intuitive but may reflect resolution of inflammatory stimuli that may have been present at clinical onset or a down-regulation of the islet-specific response in general once islet antigens are lost.

In the present review, other circulating miRNAs were associated with progression to T1D. Dysregulation in miRNA pathways including *miR-142-3p*, *miR-181a*, *mirR-181c*, *miR-193, miR-200*, *miR-27a*, *miR-29a*, *miR-330-3p* and *miR-326* may contribute to the loss of Treg function (49–52). In addition, *miR-181a-5p* from activated CD4^+^ T cells targets *SHP-2* and *PTPN22*, two genes implicated in the progression to T1D (53). Despite the many unanswered questions relating to circulating miRNAs, they are likely to provide important clues in understanding T1D progression and may be important future therapeutic targets. Interestingly, the expression of transcription factors and cytokine data reported in the various studies included in this review were often discordant, possibly due to differences in methodology.

This review is not without limitations, as selected studies were restricted to participants less than 20 years of age, and therefore does not directly address progression to T1D in adult subjects. Most of the studies in this review involved participants with high genetic risk factors and this may not be representative of the current population of individuals diagnosed with T1D in the clinical setting. Several molecular studies were excluded due to the small sample size and these articles may have provided further insight into the molecular mechanisms underlying islet autoimmunity and progression to T1D. As previously mentioned, subject selection differed for the various studies, potentially contributing to some of the discrepant findings. Variation in the attributes of the at-risk populations studied including family history of T1D and the presence or absence of certain HLAs significantly influences the risk profile.

This review highlights a number of knowledge gaps and suggests five main areas of future research. Firstly, additional research is required in the stratifying individuals at high risk of progressing to T1D. Secondly, the discrepancy in some studies suggests that the exact mechanistic pathways underlying to progression to T1D may vary. Thirdly, numerous miRNAs were identified in the review but the exact role of many of these miRNAs is yet to be determined and further studies specifically focused on circulating or cell derived miRNA may be useful in validating these molecules in other cohorts. Fourthly, given that there were only a handful of studies on the role of proteins and lipids in progression to T1D further research is required in this area. A focus on molecules likely perform better as biomarkers and be adaptable to clinical assays such as proteins or simple gene expression on whole blood should be prioritised. Lastly, the pathways responsible for progressing to early and late onset T1D are needed.

In conclusion, the included studies confirm disturbances in the prediabetes stage of T1D, particularly at the immunological and molecular level. A greater understanding of these parameters may provide future therapeutic opportunities. Future research should consider and address specific applications of these biomarkers using animal models and large samples of participants at risk of and progressing to T1D. Such research should be sufficiently powered, focused on specific cell types, and consider incorporating multiple reported biomarkers in developing prediction algorithms.

## Data availability statement

The original contributions presented in the study are included in the article/**Supplementary Material**. Further inquiries can be directed to the corresponding author.

## Author contributions

EH-W, MH and EB designed the study. EB managed data collection. EB and EH-W conducted the analysis. EH-W, MH and EB contributed to the interpretation of the results. EB wrote the initial draft of the manuscript, with revisions by all authors. The final manuscript was approved by all authors. EB, EH-W and MH are the guarantors of this work. All authors contributed to the article and approved the submitted version.
