# Supplementary material for: Circulating biomarkers during progression to type 1 diabetes: A systematic review
Source: Front Endocrinol (Lausanne). 2023 Feb 3;14:1117076. doi: 10.3389/fendo.2023.1117076 (PMC9935596; doi:10.3389/fendo.2023.1117076)
Supplement: Supplementary file 1 [file DataSheet_1.pdf]

Search Methods

Search strategy for PubMed and EBSCO ((("Type 1 Diabetes"[Title]) AND (“prediction”[title/text word])) NOT (("Mice"[title/text word]))) NOT (“Animals”[title/text word]) NOT ("reviews"[title/text word]))) NOT (“clinical trials”))), ((("Type 1 Diabetes"[Title])) AND (“predict” [title/text word])))) NOT (("Mice"[title/text word]))) NOT (“Animals” [title/text word]))) NOT ("reviews"[title/text word]))) NOT (“clinical trials” [title/text word]))))), ((("Type 1 Diabetes"[Title])) AND (“predictor” [title/text word])))) NOT (("Mice"[title/text word]))) NOT (“Animals” [title/text word]))) NOT ("reviews"[title/text word]))) NOT (“clinical trials” [title/text word]))))), ((("Type 1 Diabetes"[Title])) AND (“predicting” [title/text word])))) NOT (("Mice"[title/text word]))) NOT (“Animals” [title/text word]))) NOT ("reviews"[title/text word]))) NOT (“clinical trials” [title/text word]))))), ((("Type 1 Diabetes"[Title])) AND (“progression” [title/text word])))) NOT (("Mice"[title/text word]))) NOT (“Animals” [title/text word]))) NOT ("reviews"[title/text word]))) NOT (“clinical trials” [title/text word]))))), ((("Type 1 Diabetes"[Title])) AND (“progressors” [title/text word])))) NOT (("Mice"[title/text word]))) NOT (“Animals” [title/text word]))) NOT ("reviews"[title/text word]))) NOT (“clinical trials” [title/text word]))))), ((("Type 1 Diabetes"[Title])) AND (“progressing” [title/text word])))) NOT (("Mice"[title/text word]))) NOT (“Animals” [title/text word]))) NOT ("reviews"[title/text word]))) NOT (“clinical trials” [title/text word]))))), ((("Type 1 Diabetes"[Title])) AND (“predictor” [title/text word])))) NOT (("Mice"[title/text word]))) NOT (“Animals” [title/text word]))) NOT ("reviews"[title/text word]))) NOT (“clinical trials” [title/text word]))))), ((("Type 1 Diabetes"[Title])) AND (“biomarker” [title/text word])))) NOT (("Mice"[title/text word]))) NOT (“Animals” [title/text word]))) NOT ("reviews"[title/text word]))) NOT (“clinical trials” [title/text word]))))), ((("Type 1 Diabetes"[Title])) AND (“biomarkers” [title/text word])))) NOT (("Mice"[title/text word]))) NOT (“Animals” [title/text word]))) NOT ("reviews"[title/text word]))) NOT (“clinical trials” [title/text word]))))), ((("Type 1 Diabetes"[Title])) AND (“autoantibodies” [title/text word])))) NOT (("Mice"[title/text word]))) NOT (“Animals” [title/text word]))) NOT ("reviews"[title/text word]))) NOT (“clinical trials” [title/text word]))))), ((("Type 1 Diabetes"[Title])) AND (“islet autoimmunity” [title/text word])))) NOT (("Mice"[title/text word]))) NOT (“Animals” [title/text word]))) NOT ("reviews"[title/text word]))) NOT (“clinical trials” [title/text word]))))), ((("Type 1 Diabetes"[Title])) AND (“susceptibility” [title/text word])))) NOT (("Mice"[title/text word]))) NOT (“Animals” [title/text word]))) NOT ("reviews"[title/text word]))) NOT (“clinical trials” [title/text word]))))), ((("Type 1 Diabetes"[Title])) AND (“prognosis” [title/text word])))) NOT (("Mice"[title/text word]))) NOT (“Animals” [title/text word]))) NOT ("reviews"[title/text word]))) NOT (“clinical trials” [title/text word]))))), ((("Type 1 Diabetes"[Title])) AND (“factors” [title/text word])))) NOT

(("Mice"[title/text word]))) NOT (“Animals” [title/text word]))) NOT ("reviews"[title/text word]))) NOT (“clinical trials” [title/text word]))))), ((("Type 1 Diabetes"[Title])) AND (“measurement” [title/text word])))) NOT (("Mice"[title/text word]))) NOT (“Animals” [title/text word]))) NOT ("reviews"[title/text word]))) NOT (“clinical trials” [title/text word]))))), ((("Type 1 Diabetes"[Title])) AND (“variables” [title/text word])))) NOT (("Mice"[title/text word]))) NOT (“Animals” [title/text word]))) NOT ("reviews"[title/text word]))) NOT (“clinical trials” [title/text word]))))), ((("Type 1 Diabetes"[Title])) AND (“characterisation” [title/text word])))) NOT (("Mice"[title/text word]))) NOT (“Animals” [title/text word]))) NOT ("reviews"[title/text word]))) NOT (“clinical trials” [title/text word]))))), ((("Type 1 Diabetes"[Title])) AND (“risk” [title/text word])))) NOT (("Mice"[title/text word]))) NOT (“Animals” [title/text word]))) NOT ("reviews"[title/text word]))) NOT (“clinical trials” [title/text word]))))), ((("Type 1 Diabetes"[Title])) AND (“disease risk” [title/text word])))) NOT (("Mice"[title/text word]))) NOT (“Animals” [title/text word]))) NOT ("reviews"[title/text word]))) NOT (“clinical trials” [title/text word]))))).

Search strategy for OVID ((("Type 1 Diabetes") AND (“prediction”)) NOT (("Mice"))) NOT (“Animals”) NOT ("reviews"))) NOT (“clinical trials”))), ((("Type 1 Diabetes")) AND (“predict”)))) NOT (("Mice"))) NOT (“Animals”))) NOT ("reviews"))) NOT (“clinical trials”))))), ((("Type 1 Diabetes")) AND (“predictor”)))) NOT (("Mice"))) NOT (“Animals”))) NOT ("reviews"))) NOT (“clinical trials”))))), ((("Type 1 Diabetes")) AND (“predicting”)))) NOT (("Mice"))) NOT (“Animals”))) NOT ("reviews"))) NOT (“clinical trials”))))), ((("Type 1 Diabetes")) AND (“progression”)))) NOT (("Mice"))) NOT (“Animals”))) NOT ("reviews"))) NOT (“clinical trials”))))), ((("Type 1 Diabetes")) AND (“progressors”)))) NOT (("Mice"))) NOT (“Animals”))) NOT ("reviews"))) NOT (“clinical trials”))))), ((("Type 1 Diabetes")) AND (“progressing”)))) NOT (("Mice"))) NOT (“Animals”))) NOT ("reviews"))) NOT (“clinical trials”))))), ((("Type 1 Diabetes")) AND (“predictor”)))) NOT (("Mice"))) NOT (“Animals”))) NOT ("reviews"))) NOT (“clinical trials”))))), ((("Type 1 Diabetes")) AND (“biomarker”)))) NOT (("Mice"))) NOT (“Animals”))) NOT ("reviews"))) NOT (“clinical trials”))))), ((("Type 1 Diabetes")) AND (“biomarkers”)))) NOT (("Mice"))) NOT (“Animals”))) NOT ("reviews"))) NOT (“clinical trials”))))), ((("Type 1 Diabetes")) AND (“autoantibodies”)))) NOT (("Mice"))) NOT (“Animals”))) NOT ("reviews"))) NOT (“clinical trials”))))), ((("Type 1 Diabetes")) AND (“islet autoimmunity”)))) NOT (("Mice"))) NOT (“Animals”))) NOT ("reviews"))) NOT (“clinical trials”))))), ((("Type 1 Diabetes")) AND (“susceptibility”)))) NOT (("Mice"))) NOT (“Animals”))) NOT ("reviews"))) NOT (“clinical trials”))))), ((("Type 1 Diabetes")) AND (“prognosis”)))) NOT (("Mice"))) NOT (“Animals”))) NOT

("reviews"))) NOT (“clinical trials”))))), ((("Type 1 Diabetes")) AND (“factors”)))) NOT (("Mice"))) NOT (“Animals”))) NOT ("reviews"))) NOT (“clinical trials”))))), ((("Type 1 Diabetes")) AND (“measurement”)))) NOT (("Mice"))) NOT (“Animals”))) NOT ("reviews"))) NOT (“clinical trials”))))), ((("Type 1 Diabetes")) AND (“variables”)))) NOT (("Mice"))) NOT (“Animals”))) NOT ("reviews"))) NOT (“clinical trials”))))), ((("Type 1 Diabetes")) AND (“characterisation”)))) NOT (("Mice"))) NOT (“Animals”))) NOT ("reviews"))) NOT (“clinical trials”))))), ((("Type 1 Diabetes")) AND (“risk”)))) NOT (("Mice"))) NOT (“Animals”))) NOT ("reviews"))) NOT (“clinical trials”))))), ((("Type 1 Diabetes")) AND (“disease risk”)))) NOT (("Mice"))) NOT (“Animals”))) NOT ("reviews"))) NOT (“clinical trials”))))).

With limits on humans, full text and English language with all duplicates removed after each search.

Supplement table 1: Quality assessment of cohort studies using CASP

	Did the study address a clearly focused issue	Was the cohort recruited in an acceptable way?	Was the exposure accurately measured to minimise bias?	Was the outcome accurately measured to minimise bias?	Have the authors identified all important confounding factors?	Have the authors taken account of the confounding factors in the design and/or analysis?	Was the follow up of subjects complete enough?	Was the follow up subjects long enough?	What are the results of the study?	How precise are the results?	Do you believe the results	Can the results be applied to the local population?	Do the results of this study fit with the other available evidence	What are the implications of this study for practice?
Harms et al. 2018	Yes	Yes	N/A	Uncertain	No	No	No	No	As stated in the main text	Very precise	Yes	Yes	Yes	Identifying biomarkers for early detection of T1D
Ihantola et al., 2018	Yes	Yes	N/A	Yes	No	No	Yes	Yes		Very precise	Yes	Yes	Yes	
Jin et al. 2014	Yes	Yes	N/A	Yes	Yes	Yes	Yes	Yes		Very precise	Yes	No	Yes	
Lamichhane et al. 2018	Yes	Yes	N/A	Yes	Yes	Yes	Yes	Yes		Very precise	Yes	Yes	Yes	
Mehdi et al. 2018	Yes	Yes	N/A	Yes	Yes	Yes	Yes	Yes		Very precise	Yes	Yes	Yes	
Nielsen et al., 2012	Yes	Yes	N/A	Yes	No	No	No	No		Very precise	Yes	Yes	Yes	
Oling et al. 2012	Yes	Yes	N/A	Yes	Yes	Yes	No	No		Very precise	Yes	Yes	Yes	
Pflueger et al. 2011	Yes	Yes	N/A	No	Yes	Yes	Yes	No		Very precise	Yes	Yes	Yes	
Reinert-Hartwall et al. 2015	Yes	Yes	N/A	No	Yes	Yes	Not stated	Not stated		Very precise	No	Yes	Yes	
Salami et al. 2018	Yes	Yes	N/A	No	Yes	Yes	Yes	No		Very precise	Yes	Yes	Yes	
Sen et al. 2020	Yes	Yes	N/A	Yes	No	No	Yes	Yes		Very precise	Yes	Yes	Yes	
Simmons et al. 2019	Yes	Yes	N/A	Yes	Yes	Yes	Yes	Yes		Very precise	Yes	Yes	Yes	
Snowhite et al. 2017	Yes	Yes	N/A	Yes	Yes	Yes	Yes	Yes		Very precise	Yes	Yes	Yes	

Vecchione et al. 2020	Yes	Yes	N/A	Yes	No	No	Yes	Yes		Very precise	Yes	Yes	Yes	
Viisanen et al. 2019	Yes	Yes	N/A	No	No	No	Yes	Yes		Very precise	Yes	Yes	Yes	
von Toerne et al. 2017	Yes	Yes	N/A	No	Yes	Yes	Yes	Yes		Very precise	Yes	Yes	Yes	
Waugh et al. 2017	Yes	Yes	N/A	No	Yes	Yes	Yes	Yes		Very precise	Yes	Yes	Yes	

Supplement table 2: Quality assessment of case control studies using CASP

	**Did the study address a clearly focused issue?**	**Did the authors use an appropriate method to answer their question?**	**Were the cases recruited in an acceptable way?**	**Were the controls selected in an acceptable way?**	**Was the exposure accurately measured to minimise bias?**	**Aside from the experimental method were the groups treated equally**	**Have the authors taken account of the potential confounding factors in the design and/or in their analysis?**	**How large was the treatment effect?**	**How precise was the estimate of the treatment effect?**	**Do you believe the results?**	**Can the results be applied to the local population?**	**Do the results of this study fit with other available evidence?**
Arif et al. 2014	Yes	Yes	Yes	Yes	N/A	Yes	Yes	N/A	N/A	Yes	Yes	Yes
Ferreira et al. 2014	Yes	Yes	Yes	No	N/A	No	No	N/A	N/A	Yes	Yes	Yes
Garavelli et al. 2020	Yes	Yes	Yes	Yes	N/A	Yes	Yes	N/A	N/A	Yes	Yes	Yes
Glisic et al. 2012	Yes	Yes	Yes	Yes	N/A	No	No	N/A	N/A	Yes	Yes	Yes
Hamari et al. 2016	Yes	Yes	Yes	Yes	N/A	Yes	No	N/A	N/A	Yes	Yes	Yes
Han et al. 2011	Yes	Yes	Yes	Yes	N/A	Yes	No	N/A	N/A	Yes	Yes	Yes
Marchand et al. 2016	Yes	Yes	Yes	Yes	N/A	Yes	No	N/A	N/A	Yes	Yes	Yes
Santos et al., 2022	Yes	Yes	Yes	Yes	N/A	Yes	Yes	N/A	N/A	Yes	Yes	Yes
Starosz et al., 2022	Yes	Yes	Yes	Yes	N/A	Yes	YEs	N/A	N/A	Yes	Yes	Yes
